# Factors associated with sexual and reproductive health (SRH) stigma among women (19−25 years) with partners in Bangladesh: a cross-sectional study

**DOI:** 10.1136/bmjph-2025-003701

**Published:** 2026-07-15

**Authors:** Nabil Ahmed Uthso, Abdullah Al Zubayer, Ikram Hossen, Raiyaan Tabassum Imita, Fahmida Hoque Rimti, Sharmina Rahman Chowdhury, Munira Sharif Supty, Zerin Afroz, Neesa Nahian Madhurja, Safayet Jamil, Mohammad Shahangir Biswas

**Affiliations:** 1 Population Health Sciences, Bristol Medical School, University of Bristol, Bristol, UK; 2 Institute of Statistical Research and Training (ISRT), University of Dhaka, Dhaka, Bangladesh; 3 Department of Sociology, University of Barisal, Barishal, Bangladesh; 4 Department of Public Health and Informatics, Jahangirnagar University, Savar, Bangladesh; 5 Chittagong Medical College Hospital, Chattogram, Bangladesh; 6 Department of Public Health, North South University, Dhaka, Bangladesh; 7 Department of Clinical Psychology, University of Rajshahi, Rajshahi, Bangladesh; 8 Department of Public Health, Daffodil International University, Dhaka, Bangladesh; 9 Department of Public and Community Health, Frontier University Garowe, Puntland, Somalia; 10 Department of Biochemistry and Biotechnology, University of Science and Technology Chittagong, Chattogram, Bangladesh

**Keywords:** Sexual Health, Sociodemographic Factors, Reproductive History

## Abstract

**Introduction:**

Sexual and reproductive health (SRH) stigma remains a major barrier to young women’s access to reproductive health information and services, particularly in settings where discussions of sexuality and reproductive rights are constrained by social and cultural norms. Evidence on the prevalence and determinants of SRH stigma among young women in Bangladesh remains limited. This study aimed to estimate the prevalence of SRH stigma and identify factors associated with SRH stigma among women aged 19–25 years with male partners in Bangladesh.

**Methods:**

We conducted a cross-sectional study and collected data through both online and offline methods. This study includes a total of 414 women (19−25 years) who reported having a male partner. We conducted univariate and bivariate analyses to explore the association between the background factors and SRH stigma. We fitted a binary logistic regression to estimate adjusted associations.

**Results:**

Among 414 participants, the overall prevalence of SRH stigma was 50.97%; almost one in two women (19−25 years) with male partners were stigmatised. Results from multivariable logistic regression showed that SRH stigma was significantly associated with several factors, for example, level of education (adjusted OR (AOR): 2.00; 95% CI 1.14 to 3.51), sexual interaction with male partners (AOR: 0.29; 95% CI 0.11 to 0.77) and history of abortion (AOR: 0.38; 95% CI 0.19 to 0.77).

**Conclusions:**

More research is necessary to comprehend the factors that lead to the stigma surrounding SRH among young Bangladeshi women. These findings may help inform interventions and policies aimed at reducing SRH stigma and improving young women’s access to reproductive health information and services.

**WHAT IS ALREADY KNOWN ON THIS TOPIC**

- Bangladeshi youths turn to online platforms and social media, particularly Facebook, to access information on sexual and reproductive health (SRH) rights. They reported fearing judgement when seeking SRH services.

**WHAT THIS STUDY ADDS**

- This study uncovers determinants of SRH stigma among Bangladeshi young women aged 19−25, including sociodemographic variables and religious beliefs, providing targeted insights for effective interventions.

**HOW THIS STUDY MIGHT AFFECT RESEARCH, PRACTICE OR POLICY**

- The study’s insights can significantly impact policy by helping policymakers design interventions that challenge societal norms and enhance women’s access to SRH information and services.

## Introduction

 Sexual and reproductive health and rights (SRHR) refer to the right of individuals to make informed decisions about their sexuality and reproductive lives while respecting the rights of others. It is a basic human right for young people to get information related to SRH and rights.^1^ Such access is essential for enabling young people to realise their full potential. Access to accurate and timely information about SRH and rights is crucial for young people to develop healthy behaviours and practise safe sex. Studies show that teenagers want to learn about SRH and rights, but they may not find the available sources of information suitable.^2^ Unfortunately, limited access to SRH and rights information causes teenagers to lack knowledge in this area, leading to early marriage, teenage pregnancy and poor decision-making related to SRH.^3^ In order to achieve equitable and sustainable development, it is crucial to protect SRH and rights.^4^ Instead of being a fundamental human right and a concern for everyone’s health, SRH and rights are rarely studied and poorly handled in developing nations. Accessing SRH rights-related services and education becomes more difficult if this element of health is ignored, which threatens a woman’s health in several ways. The SRH of young people is closely tied to their overall health and can significantly impact their well-being.^5^ The incidence of several SRH issues, including sexually transmitted infections, unintended pregnancy and unsafe abortion, is increasing due to higher rates of unprotected sexual activity and limited access to reproductive health information and services.^6 7^ Young people’s need for SRH services is often overlooked.^8^ According to the study of Muntean *et al*,^9^ there is a difference between people’s knowledge, use of services and ability to access reproductive health services. Young people often receive inaccurate and insufficient information about reproductive health due to the delicate nature of the subject. In addition, cultural norms in developing nations can make it difficult for young people to discuss sexual topics with their parents openly.^10 11^ A study conducted among students aged 19−25 in Bangladesh reported that youth prefer online platforms to get information about SRH and rights knowledge. They expressed their fear of judgement by family members or physicians while seeking SRH services.^12^

According to some, stigma is a key social factor influencing health and a cause of health disparities.^13 14^ Stigma is viewed as a quality that profoundly diminishes people and turns them from complete, regular humans into tainted, devalued ones.^15^ Stigmatisation is a complicated, contextual and dynamic social process that refers to the denigration of a person for a trait that violates societal norms and is undervalued by the greater culture.^13 15^ Measures to combat stigma surrounding reproductive health have specifically targeted HIV/AIDS and abortion. There are underlying components of stigma that have been found by some validated instruments.^16 17^ But the measures of previous studies do not reflect stigma covering all significant SRH dimensions, such as sex, pregnancy, childbirth and family planning. Bangladesh, a densely populated country in South Asia, faces challenges related to SRH stigma despite improvements in healthcare infrastructure and services.^18 19^ Deeply rooted social norms, cultural beliefs and traditional values often discourage open discussions about sexuality, menstruation, contraception, premarital relationships, abortion and reproductive rights.^20 21^ In many communities, conversations regarding SRH are considered socially inappropriate, particularly for young, unmarried women.^22 23^ Fear of social judgement, family disapproval and damage to personal or family reputation may prevent women from seeking SRH-related information and healthcare services.^12^ These cultural and societal expectations contribute to stigma surrounding SRH issues and may negatively affect women’s autonomy, health-seeking behaviour and access to reproductive healthcare services.^24^ Many young women in Bangladesh encounter obstacles in obtaining accurate information and accessing necessary SRH services, leading to adverse health outcomes and compromised agency.^12^ To achieve the Sustainable Development Goal 3.7 by 2030, Bangladesh must guarantee that all citizens can access SRH services, including family planning and integrating SRH into national strategies and programmes. Therefore, this study aimed to estimate the prevalence of SRH stigma and identify the associated sociodemographic, psychosocial and reproductive health-related factors among women aged 19–25 years with male partners in Bangladesh.

## Methods

### Study design and setting

A cross-sectional study was conducted between March 2025 and May 2025 among women aged 19–25 in Bangladesh. Participants were eligible if they were women within this age range and had a current or past male partner. We employed a mixed-mode data collection strategy, incorporating both online and offline survey approaches to ensure broader participation and inclusivity, especially among women with limited internet access.

### Participant recruitment and sampling

For the online component, participants were recruited using non-probability convenience sampling. A secure survey link was distributed through popular social media platforms (eg, Facebook, WhatsApp), youth networks and digital community groups. Interested participants accessed and completed a confidential, self-administered survey using a semi-structured questionnaire hosted online.

For the offline component, trained female data collectors visited youth-friendly centres, community-based organisations and selected educational and health institutions in both urban and semi-urban settings.

These sites were selected to facilitate access to young women within the target age group while maintaining privacy and confidentiality when discussing sensitive SRH topics. The offline recruitment sites included youth-friendly health service centres, private tutoring centres attended by university and college students, community-based women’s organisations and outpatient waiting areas of non-specialised clinics where young adults frequently seek general health services. Data collectors approached potential participants individually, briefly explained the purpose of the study and screened for eligibility based on age and relationship status.

A minimum sample size of 384 was estimated based on the assumption of 50% prevalence of SRH stigma, 95% confidence level and 5% margin of error using this formula: $n=\frac{Z^{2}p(1-p)}{e^{2}}$. A total of 414 respondents were ultimately included, exceeding the minimum required for adequate power. Because recruitment involved open online distribution and voluntary participation through social media and community networks, the total number of individuals who viewed the survey invitation could not be determined. Therefore, a precise response rate could not be calculated.

### Data collection procedure

Informed consent was obtained electronically through a required digital consent form before participants could proceed with the questionnaire. Participation was voluntary, and surveys were completed in a private space to minimise social desirability bias. To ensure consistency between online and offline data collection, the same semi-structured questionnaire was used in both formats, and data collectors received standardised training on recruitment procedures, consent protocols and confidentiality practices. Responses collected using paper questionnaires were subsequently entered into the electronic dataset by trained research staff using double-entry verification to minimise data entry errors. The questionnaire was constructed in Bangla (the official language of Bangladesh) and later translated into English for data analysis. Data collectors received training on study objectives and primary data collection techniques, and a pretest was conducted before final data collection.

### Questionnaire, cultural adaptation and validation of the stigma scale

On enrolment, participants completed a confidential survey using a semi-structured questionnaire containing a 20-item stigma scale adapted from previously validated instruments developed by Hall *et al* and related studies measuring SRH stigma among young adults.^25^,^27^

The scale captures three domains of stigma: internalised stigma, enacted stigma and stigmatising lay attitudes. Participants responded to each item using a 3-point Likert scale (agree, neutral, disagree).

Minor wording modifications were made to ensure that items reflected commonly understood terminology related to SRH in Bangladesh while preserving the conceptual meaning of the original scale. Attention was given to items referring to premarital relationships, family planning, pregnancy and abortion, which may carry culturally specific connotations in the Bangladeshi social context. The revised Bangla version was then pilot-tested among a small group of women aged 19–25 (n=20) to evaluate comprehension, acceptability and item clarity. Feedback from the pilot participants resulted in minor adjustments to wording but no substantive changes to the structure of the scale.

The final adapted instrument consisted of 20 items measuring three domains of stigma: internalised stigma, enacted stigma and stigmatising lay attitudes. The full list of items used in the questionnaire is provided in online supplemental file 1.

Internal consistency was assessed using Cronbach’s alpha. The 20-item stigma scale demonstrated excellent reliability (α=0.892).

Construct validity was evaluated using exploratory factor analysis with principal-component extraction. The Kaiser-Meyer-Olkin measure of sampling adequacy was 0.883, indicating that the data were highly suitable for factor analysis. Five factors with eigenvalues greater than one were retained, explaining 64.2% of the total variance. Factor loadings supported a multidimensional structure aligning with theoretical domains.

### Outcome and exposure variables

The primary outcome variable was SRH stigma. A total stigma score ranging from 0 to 20 was generated by summing the dichotomised responses to the 20 stigma items. Participants with scores above the mean stigma score (11.46) were classified as ‘stigmatised’, whereas those scoring at or below the mean were classified as ‘not stigmatised’. Dichotomisation was performed to facilitate interpretation in logistic regression analyses and has been used in previous population-based stigma research.

The exposure variables included a range of sociodemographic factors (eg, age, area of residence, division, religion, education level, family income), religiosity indicators (religious importance and attendance), SRH history (eg, ever had sex with male partner, experience of physical or sexual abuse, ever pregnant, contraceptive use, abortion history, number of children) and health and psychosocial factors (eg, overall health rating, presence of chronic conditions, and symptoms of depression, anxiety and stress). These variables were selected based on prior literature and theoretical relevance to SRH stigma.

### Statistical analysis

We conducted univariate and bivariate analyses to describe participant characteristics and assess associations between background factors and SRH stigma. Variables considered included sociodemographic characteristics, religiosity indicators, SRH history and health and psychosocial factors. Binary logistic regression was used to examine factors associated with SRH stigma. Variables were selected for inclusion in the multivariable model based on theoretical relevance and evidence from prior literature related to SRH stigma. Adjusted and unadjusted ORs with 95% CIs were reported. Two-tailed statistical tests with p values <0.05 were considered statistically significant. Statistical analyses were conducted using Stata V.14.0 (College Station, Texas, USA).

Prior to analysis, the dataset was checked for completeness and consistency. Paper-based survey responses were entered into the electronic dataset and verified using double-entry comparison to minimise transcription errors. Data cleaning procedures included range checks and verification of logical consistency between related variables. No personally identifiable information was collected, and all data were analysed in anonymised form.

### Patient and public involvement

No patients or members of the public were involved in the design, conduct, reporting or dissemination plans of this research.

## Results

Table 1 shows that the mean age of respondents is 22.90 (SD: 1.82) years. Most (68.60%) of the respondents are from urban areas. About 85% of the respondents are Muslims. A considerable proportion (62.56%) of the participants have an education level of ‘higher secondary+’. About 27% of the respondents’ monthly family income is below or equal to 20 000 BDT, while only 18.60% have a family income (monthly) of more than 50 000 BDT. Furthermore, nearly 16% of women have been sexually assaulted, and 31.88% have been physically abused by their male partners. A significant portion (37.44%) of the respondents never received any family planning services, and 24.15% of women never used contraceptives. About 15% of women have an abortion history in their lives. Almost 12% of the participants have chronic health conditions. The mean scores of depression, anxiety and stress symptoms are 2.64, 2.99 and 3.21, respectively.

**Table 1:** Distribution of women aged 19−25 with partners by background characteristics

Variable	Levels	Frequency/mean (SD)	Percentage
Age		22.90 (1.82)	
Area	Urban	284	68.6
	Rural	130	31.4
Division	Dhaka	176	42.51
	Others	238	57.49
Religion	Islam	353	85.27
	Others	61	14.73
Education	Up to secondary	155	37.44
	Higher secondary+	259	62.56
Monthly family income (in BDT)	≤20 000	112	27.05
	20 001–30 000	94	22.71
	30 001–40 000	67	16.18
	40 001–50 000	64	15.46
	>50 000	77	18.6
Religious importance	Extremely/very important	192	46.38
	Important/somewhat important/not at all important	222	53.62
Religious attendance	At least weekly	275	66.43
	Less than weekly	139	33.57
Ever had sex with male partner	Yes	379	91.55
	No	35	8.45
Have been sexually assaulted by partner	Yes	66	15.94
	No	348	84.06
Have been physically abused by partner	Yes	132	31.88
	No	282	68.12
Ever received any family planning services	Yes	259	62.56
	No	155	37.44
Ever used any contraceptive method	Yes	314	75.85
	No	100	24.15
Pregnancy status	Ever pregnant	183	44.2
	Never pregnant	231	55.8
Ever had an abortion	Yes	63	15.22
	No	351	84.78
Have children	Yes	147	35.51
	No	267	64.49
Overall health rating	Excellent/very good	54	13.04
	Good/fair/poor	360	86.96
Presence of chronic health condition	Yes	48	11.59
	No	366	88.41
Depression symptoms*		2.64 (1.14)	
Anxiety symptoms*		2.99 (1.17)	
Stress symptoms*		3.21 (1.24)	
Overall		414	100

* Measured in 5-point Likert scale (1=never/not at all, 2=only 1–2 times per month/almost never, 3=3–4 times per month/sometimes, 4=at least once a week/fairly often, 5=almost every day/very often).

Table 2 represents that the overall prevalence of SRH stigma is 50.97% among women aged 19–25. Almost 59% of women in Dhaka are stigmatised, while 45.38% of women in other divisions were stigmatised (p value: 0.008). About 53% of Muslim women have SRH stigma, while 36.07% of women with a religious status other than Islam are stigmatised (p value: 0.012). Results show that 58.06% of women with an education level up to secondary are stigmatised, and 46.72% of women with education beyond the secondary level are stigmatised (p value: 0.025). Nearly 61% of women whose male partners abused them physically have SRH stigma, while this percentage is 46.45% for those women who have not been physically abused (p value: 0.007). Regarding the variable religious attendance, almost 44% of women who attend religious activities less than weekly are stigmatised, and about 55% of women who attend religious activities at least weekly are stigmatised (p value: 0.040).

**Table 2:** Percentage distribution of stigmatised women by background characteristics with p value

Variable	Levels	Frequency (%)	P value
Overall		211 (50.97)	
Age		22.90 (mean)	0.919
Area	Urban	150 (52.82)	0.266
	Rural	61 (46.92)	
Division	Dhaka	103 (58.52)	0.008*
	Others	108 (45.38)	
Religion	Islam	189 (53.54)	0.012*
	Others	22 (36.07)	
Education	Up to secondary	90 (58.06)	0.025*
	Higher secondary+	121 (46.72)	
Monthly family income (in BDT)	≤20 000	55 (49.11)	0.753
	20 001–30 000	53 (56.38)	
	30 001–40 000	31 (46.27)	
	40 001–50 000	32 (50.00)	
	>50 000	40 (51.95)	
Religious importance	Extremely/very important	104 (54.17)	0.226
	Important/somewhat important/not at all important	107 (48.20)	
Religious attendance	At least weekly	150 (54.55)	0.040*
	Less than weekly	61 (43.88)	
Ever had sex with male partner	Yes	189 (49.87)	0.141
	No	22 (62.86)	
Have been sexually assaulted by partner	Yes	40 (60.61)	0.088
	No	171 (49.14)	
Have been physically abused by partner	Yes	80 (60.61)	0.007*
	No	131 (46.45)	
Ever received any family planning services	Yes	130 (50.19)	0.684
	No	81 (52.26)	
Ever used any contraceptive method	Yes	161 (51.27)	0.824
	No	50 (50.00)	
Pregnancy status	Ever pregnant	97 (53.01)	0.46
	Never pregnant	114 (49.35)	
Ever had an abortion	Yes	25 (39.68)	0.052
	No	186 (52.99)	
Have children	Yes	81 (55.10)	0.212
	No	130 (48.69)	
Overall health rating	Excellent/very good	24 (44.44)	0.304
	Good/fair/poor	187 (51.94)	
Presence of chronic health condition	Yes	24 (50.00)	0.887
	No	187 (51.09)	
Depression symptoms		2.64 (mean)	0.936
Anxiety symptoms		2.99 (mean)	0.554
Stress symptoms		3.21 (mean)	0.742

* p value <0.05

Table 3 shows the binary logistic regression model output regressing the factors (demographic, health and well-being, sexual history) on the variable SRH stigma. For the variable religion, Muslim women have higher odds (adjusted OR (AOR): 1.97, 95% CI 1.00 to 3.11) of being stigmatised than women of other religions holding other variables constant. Muslim women are more likely to be stigmatised than those with a religious status other than Islam. Results show that women with education level up to secondary have two times (AOR: 2.00, 95% CI 1.14 to 3.51) higher odds of being stigmatised compared with women whose education level is higher secondary+ holding other variables constant, meaning that women who studied up to secondary level are more likely to be stigmatised. Women who have ever had sex with their partners are 71% (AOR: 0.29, 95% CI 0.11 to 0.77) less likely to be stigmatised than those who never had sex. Moreover, women whose partners abused them physically have 77% (AOR: 1.77, 95% CI 1.00 to 3.11) higher odds of having SRH stigma than those who have never been physically abused. Results also show that women with a history of abortion have 62% (AOR: 0.38, 95% CI 0.19 to 0.77) lower odds of being stigmatised than those with no history of abortion during their lifetime.

**Table 3:** Results of binary logistic regression regressing background factors on stigma showing odds ratios (adjusted and unadjusted), 95% CI and p value

Variable	Levels	Unadjusted		Adjusted	
		OR (95% CI)	P value	OR (95% CI)	P value
Age		1.01 (0.90 to 1.12)	0.918	1.08 (0.95 to 1.24)	0.23
Area	Urban	1.27 (0.84 to 1.92)	0.266	1.00 (0.57 to 1.77)	0.992
	Rural	(Reference)		(Reference)	
Division	Dhaka	1.69 (1.15 to 2.52)	0.008*	1.37 (0.82 to 2.29)	0.233
	Others	(Reference)		(Reference)	
Religion	Islam	2.04 (1.16 to 3.59)	0.013*	1.97 (1.00 to 3.11)	0.048*
	Others	(Reference)		(Reference)	
Education	Up to secondary	1.58 (1.06 to 2.36)	0.026*	2.00 (1.14 to 3.51)	0.016*
	Higher secondary+	(Reference)		(Reference)	
Monthly family income (in BDT)	≤20 000	(Reference)		(Reference)	
	20 001–30 000	1.34 (0.77 to 2.32)	0.298	1.51 (0.81 to 2.81)	0.194
	30 001–40 000	0.89 (0.49 to 1.64)	0.713	1.08 (0.54 to 2.15)	0.826
	40 001–50 000	1.04 (0.56 to 1.92)	0.909	1.49 (0.72 to 3.12)	0.281
	>50 000	1.12 (0.63 to 2.00)	0.701	1.62 (0.78 to 3.39)	0.199
Religious importance	Extremely/very important	1.27 (0.86 to 1.87)	0.226	1.24 (0.78 to 1.97)	0.357
	Important/somewhat important/not at all important	(Reference)		(Reference)	
Religious attendance	At least weekly	1.53 (1.02 to 2.31)	0.041*	1.40 (0.83 to 2.36)	0.205
	Less than weekly	(Reference)		(Reference)	
Ever had sex with male partner	Yes	0.59 (0.29 to 1.20)	0.145	0.29 (0.11 to 0.77)	0.012*
	No	(Reference)		(Reference)	
Have been sexually assaulted by partner	Yes	1.59 (0.93 to 2.72)	0.089	1.50 (0.74 to 3.05)	0.253
	No	(Reference)		(Reference)	
Have been physically abused by partner	Yes	1.77 (1.16 to 2.69)	0.008*	1.77 (1.00−3.12)	0.048*
	No	(Reference)		(Reference)	
Ever received any family planning services	Yes	0.92 (0.62 to 1.37)	0.684	0.98 (0.60 to 1.61)	0.959
	No	(Reference)		(Reference)	
Ever used any contraceptive method	Yes	1.05 (0.67 to 1.65)	0.824	1.46 (0.79 to 2.71)	0.227
	No	(Reference)		(Reference)	
Pregnancy status	Ever pregnant	1.16 (0.79 to 1.71)	0.46	1.37 (0.57 to 3.29)	0.482
	Never pregnant	(Reference)		(Reference)	
Ever had an abortion	Yes	0.58 (0.34 to 1.01)	0.053	0.38 (0.19 to 0.77)	0.007*
	No	(Reference)		(Reference)	
Have children	Yes	1.29 (0.86 to 1.94)	0.212	0.81 (0.34 to 1.94)	0.641
	No	(Reference)		(Reference)	
Overall health rating	Excellent/very good	0.74 (0.42 to 1.32)	0.305	0.71 (0.36 to 1.40)	0.326
	Good/fair/poor	(Reference)		(Reference)	
Presence of chronic health condition	Yes	0.96 (0.52 to 1.75)	0.887	0.71 (0.34 to 1.47)	0.355
	No	(Reference)		(Reference)	
Depression symptoms		1.00 (0.85 to 1.19)	0.935	0.89 (0.67 to 1.17)	0.395
Anxiety symptoms		1.05 (0.89 to 1.24)	0.553	1.13 (0.84 to 1.54)	0.422
Stress symptoms		1.03 (0.88 to 1.19)	0.741	0.97 (0.75 to 1.27)	0.849

* p value <0.05.

## Discussion

We discovered an association between respondents’ religion and SRH stigma. Lack of understanding about contraception and basic reproductive knowledge contributes to low contraceptive usage among Muslim women.^28^,^30^ Many women are misled into thinking that Islam forbids contraception since the use of permanent measures of contraception is not permitted unless the health or life of the expecting mother is in danger.^31^ These factors may contribute to stronger stigmatising attitudes surrounding SRH issues in certain communities. However, the present study found that women’s education level was significantly related to their stigmatisation. A study in South Korea showed that boosting young women’s reproductive health behaviour requires considering sexual perspectives and sexual literacy.^32^ Another US study showed that increased victimisation, adolescent pregnancies and dropout rates result from a lack of sex education policy.^33^ A survey conducted in Kenya found that in sub-Saharan Africa, the association between a woman’s level of education and her sexually related behaviours is best explained by gender inequality and a lack of information.^34^ Therefore, women with lower levels of education are less likely to have access to sex education and effective family planning methods, increasing the SRH stigma they face. A significant association was also found between sexual experience with a male partner and stigma, which is consistent with the finding of Hall *et al*.^26^ Moreover, a significant association between being physically abused by a partner and stigma was found. Women who have experienced physical abuse may be less likely to disclose their experiences because of fear of social judgement, stigma or further violence. The primary causes of this silence were high acceptance of violence, stigma and fear of more injury.^35^ An association was also observed between abortion history and SRH stigma. In the adjusted analysis, women who reported a history of abortion had significantly lower odds of being classified as stigmatised compared with those who had never had an abortion. This finding should be interpreted with caution, as abortion is widely recognised as a highly stigmatised reproductive experience in many social and cultural contexts, including South Asia. Importantly, the stigma measure used in this study captures perceived and internalised stigma related to a broader set of SRH issues rather than stigma directed specifically towards abortion. Therefore, the observed association should not be interpreted as indicating that abortion itself is socially less stigmatised in the Bangladeshi context.

Several plausible explanations may account for this pattern. Women who have experienced abortion may have had greater interaction with reproductive health services and providers, which could increase their exposure to accurate SRH information and reduce internalised stigma related to reproductive health topics more broadly. Previous research suggests that engagement with SRH services can improve reproductive health knowledge and confidence in navigating sensitive health issues. Another possible explanation is that women willing to disclose abortion experiences in a survey may represent a subgroup with comparatively lower internalised stigma or greater autonomy in reproductive decision-making. Additionally, women who have previously managed unintended pregnancies may develop greater familiarity with reproductive health systems and support networks, which could influence their perceptions of stigma. Given the sensitive nature of abortion reporting and the cross-sectional design of the study, further research is needed to better understand the mechanisms underlying this association in the Bangladeshi context. However, a study conducted in Western Kenya revealed that students had experienced stigma related to abortion and the use of contraceptives.^36^ In recent research in the USA, two-thirds of women said they felt judged by others if they were open about having an abortion, and more than half said they had to hide the procedure from loved ones.^37^

Our research suggests that young women in Bangladesh experience and accept stigmatising views on SRH issues. These findings highlight the importance of addressing both structural and interpersonal sources of stigma that influence young women’s access to SRH information and services. Some young women may be more vulnerable due to their life experiences. While our results shed light on the factors contributing to SRH stigma, they also reveal the need for further research to develop evidence-based strategies to fight SRH stigma among young women. This study aims to provide insights into the multifaceted determinants perpetuating SRH stigma among Bangladesh’s young women. By understanding these factors, this research hopes to contribute to developing evidence-based strategies to improve SRH outcomes and women’s empowerment in the country and beyond.

### Limitations of the study

Several limitations should be considered when interpreting the findings of this study. First, the cross-sectional design limits the ability to establish causal relationships between background characteristics and SRH stigma. The associations observed should therefore be interpreted as correlational rather than causal. Second, the use of non-probability convenience sampling and partial online recruitment may introduce selection bias. Individuals who are active on social media or more willing to participate in online surveys may differ systematically from those who are less digitally connected or less comfortable discussing SRH topics. Although the study also incorporated offline recruitment to broaden participation, the sample may still over-represent relatively educated and urban young women, which may limit the generalisability of the findings to all young women in Bangladesh. Third, the study relied on self-reported information for sensitive experiences such as sexual activity, abortion and partner abuse. These topics may be subject to social desirability bias or under-reporting, particularly within conservative social contexts where discussing reproductive health matters can be socially sensitive. As a result, some behaviours or experiences may have been misreported or under-reported. Fourth, the SRH stigma measure was operationalised as a dichotomised index based on the mean score of the stigma scale. While this approach facilitated interpretation in regression analyses, dichotomising a multidimensional construct such as stigma may reduce variability in responses and potentially obscure more nuanced differences in stigma experiences. Finally, because the study focused on women aged 19–25 who reported having a male partner, the findings may not reflect the experiences of younger adolescents, unmarried women without partners or other populations who may face different forms of SRH-related stigma.

### Conclusion

This study found that SRH stigma is highly prevalent among women aged 19–25 years with male partners in Bangladesh, with approximately one in two participants classified as experiencing SRH stigma. SRH stigma was significantly associated with several sociodemographic and reproductive factors, including religion, educational attainment, sexual experience with a male partner, experiences of physical abuse and abortion history.

These findings highlight the complex social and cultural factors that shape young women’s perceptions and experiences of SRH in Bangladesh. Efforts to reduce SRH stigma should focus on improving access to accurate reproductive health information, strengthening comprehensive sexuality education, addressing gender-based violence and promoting supportive environments for young women to seek SRH information and services without fear of judgement or discrimination.

Further research using longitudinal and nationally representative study designs is needed to better understand the pathways through which social, cultural and reproductive experiences influence SRH stigma and to inform the development of effective stigma-reduction interventions and policies in Bangladesh.

## Supplementary material

10.1136/bmjph-2025-003701 online supplemental file 1

## Data Availability

Data are available upon reasonable request.
