# Supplementary material for: Factors associated with sexual and reproductive health (SRH) stigma among women (19−25 years) with partners in Bangladesh: a cross-sectional study
Source: BMJ Public Health. 2026 Jul 15;4(3):e003701. doi: 10.1136/bmjph-2025-003701 (PMC13374404; doi:10.1136/bmjph-2025-003701)
Supplement: online supplemental file 1 [file bmjph-4-3-s001.docx]

Questionnaire

**Factors associated with sexual and reproductive health (SRH) stigma among women (19−25 years) with partners in Bangladesh: A cross-sectional study**

This study aims to explore the factors associated with sexual and reproductive health stigma among women (19−25 years) in Bangladesh. This questionnaire includes some socio-demographic, health & social wellbeing and sexual & reproductive history-related questions. Your participation in this survey is completely anonymous. That is, we do not require your name or email address.

**Do you want to participate in this survey? 1=Yes 2=No**

**Part 1: Socio-demographic characteristics**

Q1. Your age (in years): ……………

Q2. Your age of residence: 1= Urban 2= Rural

Q3. Your division: 1= Dhaka 2= Mymensingh

3= Rangpur 4= Rajshahi

5= Khulna 6= Sylhet

7= Barishal 8= Chattogram

Q4. Your religion: 1= Islam 2= Hindu

3= Christian 4= Buddhist

5= Other

Q5. Ethnicity: 1= Bengali 2= Other

Q6. Monthly income of your family (in BDT):

1= <10,000 2= 10,000-20,000

3= 20,001-30,000 4= 30,001-40,000

5= 40,001-50,000 6= >50,000

Q7. Education: 1= No formal education

2= Primary or pre-primary

3= Secondary 4= Higher secondary+

Q8. Your marital status: 1= Unmarried 2= Married

3= Divorced/ Separated 4= Widowed

Q9. Your religious attendance:

1= At least once a week

2= At least once a month

3= Less than monthly

Q10. Is religious activity important to you?

1= Not at all

2= Somewhat important

3= Important

4= Very important

5= Extremely important

**Part 2: Health and social well-being**

Q11. Is religious activity important to you?

1= Poor

2= Fair

3= Good

4= Very good

5= Excellent

Q12. Do you have any depression symptoms?

1= Never/ Not at all

2= Only 1-2 times per month/ Almost never

3= 3-4 times per month/ Sometimes

4= At least once a week/ Fairly often

5= Almost every day/ Very often

Q13. Do you have any anxiety symptoms?

1= Never/ Not at all

2= Only 1-2 times per month/ Almost never

3= 3-4 times per month/ Sometimes

4= At least once a week/ Fairly often

5= Almost every day/ Very often

3= Badly

Q14. Do you have any stress symptoms?

1= Never/ Not at all

2= Only 1-2 times per month/ Almost never

3= 3-4 times per month/ Sometimes

4= At least once a week/ Fairly often

5= Almost every day/ Very often

Part 3: Sexual and Reproductive History

Q15. Do you have any male partners? 1= Yes 2= No

Q16. Ever had sex with your male partner? 1= Yes 2= No

Q17. Do you have been sexually assaulted by your partner? 1= Yes 2= No

Q18. Do you have been physically abused, partner? 1= Yes 2= No

Q19. Ever received any family-planning services? 1= Yes 2= No

Q20. Ever used any contraceptive method? 1= Yes 2= No

Q21. Pregnancy status: 1= Yes 2= No

Q22. Do you have any abortions in your life? 1= Yes 2= No

Q23. Do you have any children? 1= Yes 2= No

**Part 4: Here are some statements. For each statement try to answer what you really think: agree (1), neutral (2), disagree (3).**

Q24. People behave differently toward a teen whom they know has had sex:

1= agree

2= neutral

3= disagree

Q25. People behave differently toward a teen whom they know has had an abortion:

1= agree

2= neutral

3= disagree

Q26. People behave differently toward a teen whom they know has used modern family-planning methods:

1= agree

2= neutral

3= disagree

Q27. Having sex as a teen often leads to getting beaten or physically hurt by one's parents:

1= agree

2= neutral

3= disagree

Q28. People behave differently toward a teen whom they know has had sex:

1= agree

2= neutral

3= disagree

Q29. Becoming pregnant and having a baby as a teen would cause people to behave differently around me:

1= agree

2= neutral

3= disagree

Q30. Becoming pregnant and having a baby as a teen would cause others to tease, insult, swear, or gossip about me:

1= agree

2= neutral

3= disagree

Q31. Having sex as a teen is a form of disobedience:

1= agree

2= neutral

3= disagree

Q32. Young women who have abortions are bad girls:

1= agree

2= neutral

3= disagree

Q33. Young women who use modern family planning are promiscuous:

1= agree

2= neutral

3= disagree

Q34. Teens who use modern family planning are viewed as bad girls:

1= agree

2= neutral

3= disagree

Q35. Having sex as a teen brings disgrace and shame to a young woman and her family:

1= agree

2= neutral

3= disagree

Q36. Becoming pregnant and having a baby as a teen would bring disgrace to my family:

1= agree

2= neutral

3= disagree

Q37. Becoming pregnant and having a baby as a teen would make me feel ashamed and bad about myself:

1= agree

2= neutral

3= disagree

Q38. Young women who have abortions will encourage others to have abortions:

1= agree

2= neutral

3= disagree

Q39. Modern family planning is not acceptable for unmarried women:

1= agree

2= neutral

3= disagree

Q40. Modern family-planning methods have bad effects on a woman's health:

1= agree

2= neutral

3= disagree

Q41. Having an abortion is committing murder:

1= agree

2= neutral

3= disagree

Q42. The media, including the television, the Internet, and magazines, has a strong impact on teens' sexual behavior:

1= agree

2= neutral

3= disagree

Q43. When teens have sex for the first time, it is because they were pressured by their friends or partners to do so:

1= agree

2= neutral

3= disagree

Q44. Children born to teen parents are worse off than those born to adults:

1= agree

2= neutral

3= disagree

**Part 5: Here are some statements. For each statement try to answer what you really think: yes (1), no (2).**

Q45. Do youths have the right that their use of reproductive health services is kept confidential?

1= yes

2= no

Q46. A man should get sex whenever he wants, irrespective of his wife’s wish:

1= yes

2= no

Q47. Does a married woman have the right to limit the number of her children according to her desire without her husband’s consent?

1= yes

2= no

Q48. Husband has no obligation to share childcare:

1= yes

2= no

Q49. Do girls have the right to resist genital mutilation against their families will?

1= yes

2= no

Q50. Do youths have a full right to access all RHSs without parents’ consent?

1= yes

2= no

Q51. Do girls have the right to autonomous reproductive choices without their partners consent?

1= yes

2= no

Q52. Do you think that all students must be free to enjoy and control their sexual and reproductive life?

1= yes

2= no

Q53. Do unmarried woman have the right to maternity leave with adequate social security benefits?

1= yes

2= no

Q54. Unmarried couples have no right to use contraceptives other than condoms:

1= yes

2= no
